# The Effect of Branched-Chain Amino Acids, Citrulline, and Arginine on High-Intensity Interval Performance in Young Swimmers

**DOI:** 10.3390/nu10121979

**Published:** 2018-12-14

**Authors:** Chun-Fang Hsueh, Huey-June Wu, Tzu-Shiou Tsai, Ching-Lin Wu, Chen-Kang Chang

**Affiliations:** 1 Graduate Institute of Sport Coaching Science, Chinese Culture University, Taipei 114, Taiwan; swim_speedo@hotmail.com; 2 Department of Combat Sports and Martial Arts, Chinese Culture University, Taipei 114, Taiwan; wuhc0123@gmail.com; 3 Taipei Municipal Nan Gang High School, Taipei 115, Taiwan; Tsai481026@gmail.com; 4 Graduate Institute of Sports and Health Management, National Chung Hsing University, Taichung 402, Taiwan; psclw@dragon.nchu.edu.tw; 5 Department of Sport Performance, National Taiwan University of Sport, 16, Section 1, Shaun-Shih Road, Taichung 404, Taiwan

**Keywords:** central fatigue, tryptophan, ammonia, nitric oxide, stroke rate, stroke count

## Abstract

High-intensity interval training has drawn significant interest for its ability to elicit similar training responses with less training volume compared to traditional moderate-intensity protocols. The purpose of this study was to examine the effect of co-ingestion of branched-chain amino acids (BCAA), arginine, and citrulline on 8 × 50 m high-intensity interval swim performance in trained young swimmers. This study used a randomized cross-over design. Eight male (age 15.6 ± 1.3 years) and eight female (age 15.6 ± 0.9 years) swimmers completed both amino acids (AA) and placebo (PL) trials. The participants ingested 0.085 g/kg body weight BCAA, 0.05 g/kg body weight arginine and 0.05 g/kg body weight citrulline before the swim test in the AA trial. The average 50 m time was significantly shorter in the AA trial than that in the PL trial. The AA trial was faster than the PL trial in the first, second, and the seventh laps. The AA trial showed significantly higher plasma BCAA concentrations and lower tryptophan/BCAA ratio. The other biochemical parameters and ratings of perceived exertion were similar between the two trials. The results showed that BCAA, arginine, and citrulline, allowed the participants to swim faster in a high-intensity interval protocol in young swimmers.

## 1. Introduction

High-intensity interval training (HIIT) has drawn significant interest from athletes of various sports, as well as general or less-fit populations [1,2]. The high-intensity nature of this protocol recruits both type-I and -II muscle fibers [3], resulting in significant improvement in cardiopulmonary and anaerobic capabilities [1,2,4,5]. Several studies have shown that HIIT can elicit similar training responses in competitive swimmers with less training volume compared to traditional moderate-intensity protocols [6,7]. One of the important factors for the success in HIIT is the ability to maintain the training intensity, especially at the later stages. However, the accumulation of peripheral and/or central fatigue, resulting from repeated high-intensity bouts, leads to declines in exercise intensity at the later stages of HIIT, and potentially reduce the training effect [1,2].

The peripheral factors for fatigue in HIIT include limitations in anaerobic and aerobic energy supply, and intramuscular accumulation of metabolic by-products such as H^+^ and inorganic phosphate [8,9]. Moreover, the central nervous system may also be involved, as the capacity of the motor cortex to drive the knee extensors after high-intensity intermittent cycling was significantly decreased [10]. One of the mechanisms that contributes to central fatigue is the increase in cerebral concentration of the neurotransmitter serotonin (5-hydroxytryptamine) during exercise. The increased cerebral serotonin could lead to the feeling of fatigue and the loss of central drive and motivation [11]. An increase in serotonin concentration in presynaptic neutrons would lead to increased serotonin release and serotonin binding to postsynaptic receptors during nerve stimulation [12]. In addition, cerebral serotonin concentration was inversely correlated to running time to fatigue in rodents [13,14]. The rate of cerebral serotonin synthesis is regulated by the transport of free tryptophan, the precursor to serotonin, across the blood brain barrier [15]. Branched-chain amino acids (BCAA) have been hypothesized to alleviate central fatigue by competing with tryptophan in crossing the blood brain barrier through the L-system transporter [16]. Indeed, the decreased plasma free tryptophan/BCAA ratio would reduce the uptake of tryptophan, and subsequently, serotonin synthesis in the brain [14].

Nitric oxide (NO), a signaling molecule with a wide range of physiological functions, has been suggested to improve exercise performance by enhancing exercise-induced vasodilation [17], increasing the oxygenation status in the working muscles, and improving VO_2_ kinetics [18]. Supplementations of arginine or citrulline, both of which are precursors to NO, have been suggested to improve performance in high-intensity exercise [19,20].

Previously, we revealed that supplementation of BCAA and arginine could improve repeated sprint performance in handball players [21]. We later added citrulline to the supplementation regimen [22,23] because the combined ingestion of citrulline and arginine may be more effective in increasing plasma arginine concentration than consuming either amino acid individually [24]. Although the nutritional strategies to support HIIT have been proposed, most results were obtained from land-based exercise [25]. In addition, the combination of BCAA, arginine, and citrulline on high-intensity interval swimming performance has not been investigated. With the increasing application of HIIT in swimming training, the aim of this study was to examine the effect of co-ingestion of BCAA, arginine, and citrulline on 8 × 50 m swim performance in trained young swimmers. The biochemical and stroke parameters were analyzed to investigate the potential mechanism of the supplementation.

## 2. Materials and Methods

### 2.1. Participants

Eight male (age: 15.6 ± 1.3 years; height: 1.74 ± 0.05 m; weight: 64.4 ± 7.6 kg) and eight female (age: 15.6 ± 0.9 years; height: 1.58 ± 0.06 m; weight: 54.0 ± 8.4 kg) swimmers were recruited from a high school in northern Taiwan. All participants have been participating in swimming training for at least 7 years and have competed at the national or international level. The characteristics and personal best performance of the participants are presented in Table 1. The exclusion criteria included cardiovascular disease risks, musculoskeletal injuries, smoking, or consumption of protein supplements in the past three months. After the experimental procedure and potential risks were explained, all participants and their legal guardians gave their written informed consent. The study protocol was approved by the Research Ethics Committee of China Medical University Hospital (CRREC-105-003).

### 2.2. Study Design

**Figure 1:**
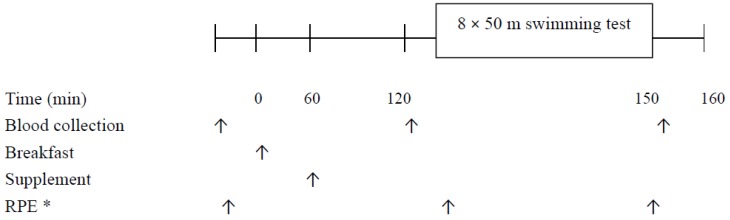
Study protocol. * RPE: ratings of perceived exertion. The symbols (↑) pointed out the actions at the specific time point.

This study used a double-blind, placebo-controlled, randomized cross-over design. The study protocol is outlined in Figure 1. Each participant completed two trials, amino acids (AA) and placebo (PL), in a random order, separated by a wash-out period of at least seven days. The regular training schedule and dietary habits were maintained during the study period. The participants refrained from all training activity on the day prior to the trial. The study was conducted in January, 2017.

### 2.3. Dietary Control

During the two days prior to each trial, the participants were provided with the same three meals per day, purchased from local convenience stores. The meals provided approximately 1624 kcal/day with 55% energy from carbohydrate, 25% from fat, and 18% from protein, according to the manufacturer’s label. A standardized breakfast was given on the days of trials, including white bread 0.5 g/kg body weight, jam 0.1 g/kg body weight, butter 0.l g/kg body weight, and soybean milk 5 mL/kg body weight (6.2 kcal/kg body weight, containing carbohydrate 1.0 g/kg body weight, protein 0.24 g/kg body weight, and fat 0.14 g/kg body weight).

### 2.4. Supplementation

On the days of the trials, the participants reported to a 25 m swimming pool in the morning after an overnight fast. After collecting blood samples from the antecubital vein, the participants consumed the standard breakfast, followed by one of the two interventions. In the AA trial, the participants ingested 0.085 g/kg body weight BCAA (leucine: isoleucine: valine = 10:7:3, containing vitamin E 6.67 IU/g BCAA, capsule, General Nutrition Corporation, Pittsburgh, PA, USA), 0.05 g/kg body weight arginine, and 0.05 g/kg body weight citrulline (arginine: citrulline = 1:1, tablet, General Nutrition Corporation). In the placebo (PL) trial, the participants consumed the identical number of empty capsules and tablets containing starch (Chung-Yu Biotech Co LTD, Taichung, Taiwan) and one capsule of vitamin E (100 IU, General Nutrition Corporation). All capsules and tablets were taken with water within 15 min. 

### 2.5. High-Intensity Interval Swimming Test

Thirty min after consuming the amino acids or placebo, the participants began a controlled 1000 m warm-up. The warm-up included 8 × 50 m easy swim focusing on individual skills, 200 m mixed style, and 4 × 100 m free style. The warm-up lasted approximately 20 min. The 8 × 50 m high-intensity interval swimming test started 60 min after consuming the amino acids or placebo, following the international rules with push starts. There was a 3-min active recovery period between each sprint. The approximate work to rest ratio of 1:6 is chosen to allow the better recovery of acid/base balance and creatine phosphate resynthesis [2]. The participants were asked to swim with their best style with the best effort in each sprint. Four male and five female participants swam front crawl, two male and three female participants swam butterfly, one male participant swam breaststroke and another male participant swam backstroke. Participants from both trials were grouped into 3 or 4 according to their best record, and competed at the same time to encourage the best performance. No food or fluid was provided during the test. The ratings of perceived exertion (RPE) were recorded immediately before and after the test using Borg’s 20-point scale [26].

### 2.6. Stroke Characteristics

The stroke characteristics were analyzed by an experienced swimmer/coach by reviewing the video files of the entire trials. The time to finish three consecutive strokes, measured by a stop watch, was recorded during approximately 35–40 m in each lap. The stroke rate was determined by dividing the time by three. For front crawl and backstroke, the timing started when the right hand entered the water, and ended immediately before the right hand entered the water for the second time. For breaststroke, the timing started when the arms started the outsweep, and ended when the arms completed the forward extension for the third time. For butterfly, the timing started when the arms entered the water, and ended immediately before the arms entered the water for the third time. The stroke count was measured during the entire lap, excluding the sculling or flipper movement after the start or turn.

### 2.7. Measurement of Blood Biochemical Parameters

The time point of blood sampling is shown in Figure 1. Venous blood samples were collected into tubes containing EDTA. After centrifugation at 1500× *g* for 15 min at 4 °C, the plasma samples were aliquoted and stored at −70 °C until further analysis. Plasma BCAA concentrations were measured enzymatically (Biovision, Milpitas, CA, USA) with a microplate spectrophotometer (Benchmark Plus, Bio-Rad, Hercules, CA, USA). Plasma tryptophan concentrations were analyzed with a fluorescence assay (Bridge-It, Mediomics, St. Louis, MO, USA). The fluorescence at excitation 485 nm and emission 665 nm was read by a microplate fluorescence reader (Plate Chameleon, Hidex, Turku, Finland). Plasma NO_x_ concentrations were determined using Griess reagent [27]. Plasma concentrations of urea, glucose, lactate, NH_3_, glycerol, and non-esterified fatty acids (NEFA) were measured enzymatically with an automatic analyzer (Hitachi 7020, Tokyo, Japan) using commercial kits (Randox, Antrim, UK). Hemoglobin concentration and hematocrit in whole blood were measured by a blood cell analyzer (Sysmex Kx-21, Diamond Diagnostics, Holliston, MA, USA) to correct potential changes in plasma volume during the study periods [28].

### 2.8. Statistical Analysis

The results were initially analyzed by three-way (gender × trial × time) analysis of variance with repeated measurements. However, gender effect was insignificant in all performance, biochemical and stroke parameters. Therefore, the data from both genders were pulled together and analyzed by two-way (trial × time) analysis of variance with repeated measurements. If the interaction effect was to be found significant, the differences between the two trials were identified by Ryan-Holm-Bonferroni post hoc analysis [29]. If the time or lap effect was to be found significant, the differences within the same trial were identified with Bonferroni post hoc analysis. A *p* < 0.05 is considered statistically significant. With the power of 0.80 and sample size of 16, the minimal detectable difference is 1.06 standard deviation (SD). The data are presented as mean ± SD.

## 3. Results

**Figure 2:**
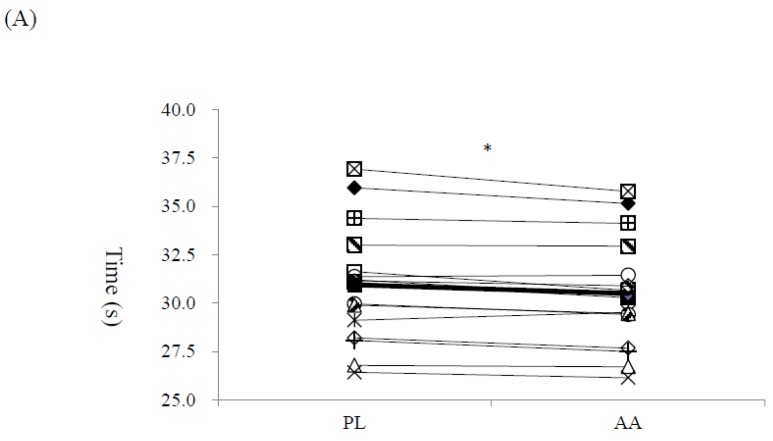

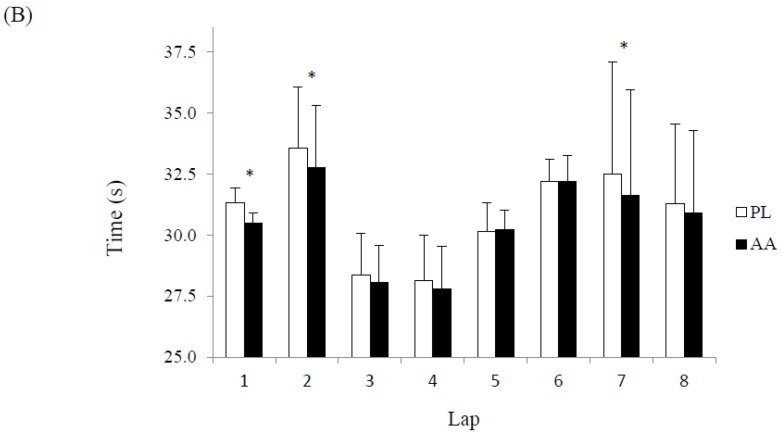
The performance of the 8 × 50 m high-intensity interval swimming test in the AA (amino acids supplemented) and PL (placebo) trials. (**A**) the average lap time of each participant.* Significant difference between the AA and PL trials, *p* < 0.05; (**B**) the average time in each lap. Trial effect: *p* < 0.001; lap effect: *p* < 0.001; interaction effect: *p* < 0.001; * Significant difference between the AA and PL trials, *p* < 0.05.

The average 50 m time of the eight laps in the high-intensity interval swimming test was significantly shorter in the AA trial than that in the PL trial (AA: 30.50 ± 2.87 s vs. PL: 30.94 ± 3.02 s, *p* < 0.001, Figure 2a). Thirteen participants (6 male and 7 female) out of total 16 had faster average lap time in the AA trial (Figure 2a). When each lap was considered separately, there were significant trial, lap, and interaction effects (all *p* < 0.001, *η*^2^ = 0.795, 0.452, 0.236, respectively). The post-hoc analysis showed that AA trial was faster than the PL trial in the first (AA: 30.47 ± 0.44 s vs. PL: 31.34 ± 0.57 s, *p* < 0.001), second (AA: 32.76 ± 2.54 s vs. PL: 33.58 ± 2.48 s, *p* < 0.001), and the seventh laps (AA: 31.64 ± 4.30 s vs. PL: 32.50 ± 4.60 s, *p* < 0.001) (Figure 2b).

**Figure 3:**
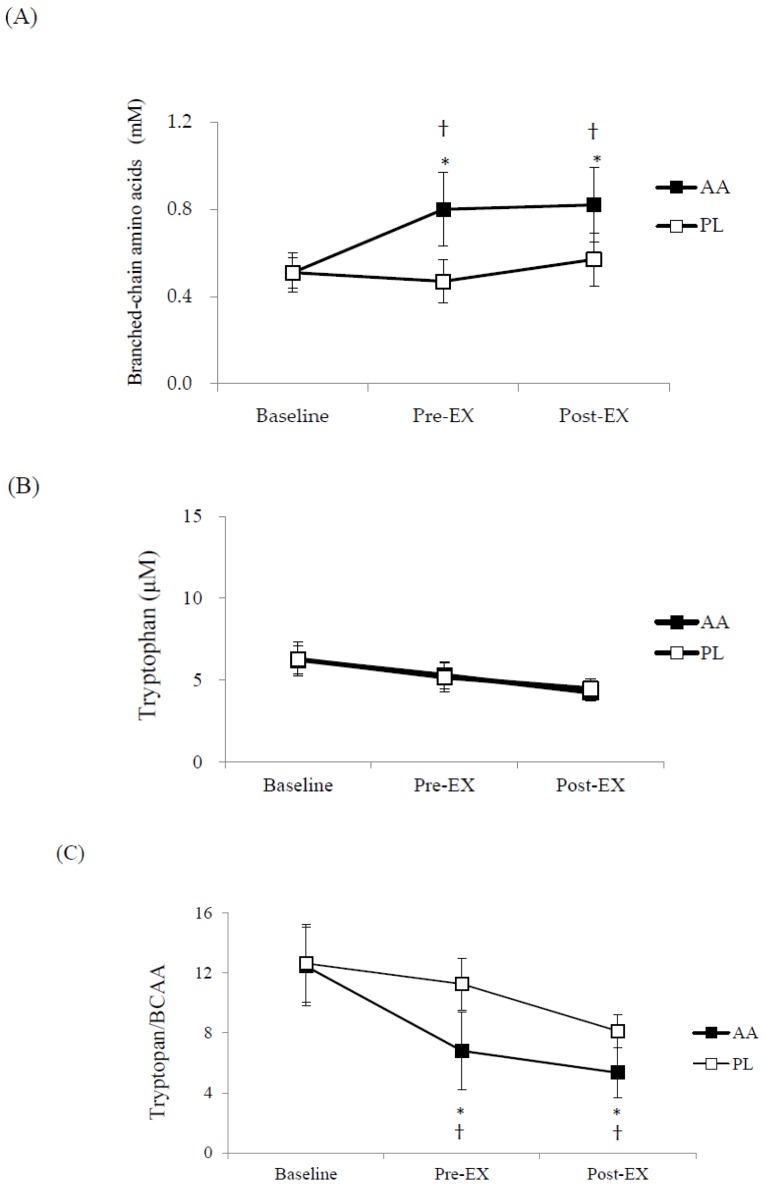
Plasma amino acid concentrations at baseline, before, and after the 8 × 50 m high-intensity interval swimming test in the AA (amino acids supplemented) and PL (placebo) trials. (**A**) Branched-chain amino acids, trial effect: *p* < 0.001; time effect: *p* < 0.001; interaction effect: *p* < 0.001. (**B**) Tryptophan, trial effect: *p* = 0.821; time effect: *p* < 0.001; interaction effect: *p* = 0.383. (**C**) Tryptophan/branched-chain amino acids ratio, trial effect: *p* < 0.001; time effect: *p* < 0.0001; interaction effect: *p* < 0.001; * Significantly different from the baseline in the same trial, *p* < 0.05; † AA trial vs. PL trial at the same time point, *p* < 0.05. Pre-Ex: before 8 × 50 m swim test; Post-Ex: immediately after 8 × 50 m swim test.

The AA trial showed significantly higher plasma BCAA concentrations before and after the 8 × 50 m test (Figure 3a). Plasma tryptophan concentrations were decreased after the interval swim in both trials at the similar magnitude (Figure 3b). Nevertheless, the elevated plasma BCAA concentrations lead to significantly lower tryptophan/BCAA ratios before and after the interval swim in the AA trial (Figure 3c). Plasma NO_x_ levels showed a significant time effect (*p* < 0.001), but the post hoc analysis did not find significant difference among the three time points in either trials (Table 2). Plasma concentrations of NH_3_, urea, lactate, glycerol, and NEFA were similar in both trials (Table 2). The participants also reported similar RPE before and after the 8 × 50 m test in both trials (Table 2).

The data of stroke rate and stroke count are shown in Table 3. None of the main effects was significant in stroke rate. However, the participants in the AA trial showed a trend of faster stroke rate in the first (*p* = 0.011) and second lap (*p* = 0.011 and 0.002, respectively, according to paired *t*-test), compared to those in the PL trial. The last lap showed the highest stroke count in both trials. In the AA trial, the participants swam the last lap with significantly higher stroke count than in the third lap (*p* = 0.015), while in the PL trial, the stroke count in the last lap was significantly higher than in the third (*p* = 0.038) and fourth lap (*p* = 0.041).

## 4. Discussion

The results of this study suggested that the combined supplementation of BCAA, arginine, and citrulline significantly improved performance in 8 × 50 m high-intensity interval swims in well-trained young swimmers. The participants in the AA trial showed significantly lower plasma tryptophan/BCAA ratio before and after the interval swims. In addition, the participants were able to swim faster under a similar degree of perceived effort, indicating the possibility of alleviated central fatigue.

The participants in the AA trial showed significantly higher plasma BCAA concentrations and lower tryptophan/BCAA ratio after the interval swims, compared to the PL trial. This is in agreement with our previous studies using the same supplements [22,23]. It has been revealed that central nervous system plays a role in the development of fatigue in repeated high-intensity exercise [9]. Several studies using functional magnetic resonance imaging have found increased activations in sensory processing and motor-related brain regions such as primary motor cortex, supplementary motor area and pre-motor cortex while performing fatiguing exercise tasks [30]. These activations suggest a greater perception of effort under fatigue and the need for higher motor output to sustain the same physical workload [31]. The decreased plasma tryptophan/BCAA ratio in the AA trial would reduce the cerebral uptake of tryptophan, leading to lower cerebral serotonin synthesis [14].

Although previous studies have reported that oral supplementation of BCAA could reduce RPE and mental fatigue in maximal exercise in untrained participants [32], our results suggest that the participants in the AA trial had better performance under the same level of RPE. This contradiction may result from the different protocols to measure physical performance. Blomstrand et al. used a fixed-rate protocol, followed by a 20-min maximal exercise on a bicycle treadmill [32]. On the other hand, the time-trial style of the present protocol required the participants to swim at their best effort in each lap. Therefore, the participants in both trials reached similar RPE, which is in agreement with our previous study [33].

A recent study revealed that a short-term citrulline supplementation could increase peak power output by 9% and total power output by 7% in 60-s all-out sprint that followed the 6-min bout of severe-intensity exercise [20]. In addition, a single dose of citrulline malate could improve performance in repeated high-intensity anaerobic resistance exercises [34]. Acute beetroot juice supplementation, which is rich in citrulline, also improved peak and mean power output, while reducing the time required to reach peak power output in the Wingate test [35]. A recent review summarized that acute beetroot juice could improve the performance in repeated high-intensity exercise by increasing phosphocreatine resynthesis and muscle shortening velocity [36]. These ergogenic effects could be mediated by the increased tissue oxygenation and improved O_2_ kinetics. In the first and second lap during which central fatigue may not have been accumulated, it is possible that ergogenic effect was partially the result of the role of citrulline in improving short-term anaerobic performance. At the later stage of repeated high-intensity exercise, the aerobic system becomes an important energy source despite the all-out effort [9]. For example, the contribution of aerobic energy increased from 25% in the first sprint to approximately 50% in the second to forth sprints in 30 s × 4 swimming with 30 s rest in between [37]. Our participants also showed significantly elevated plasma glycerol concentrations after 8 × 50 m swim (Table 2), indicating greater lipolysis. The increased phosphocreatine resynthesis and muscle work efficiency, combining with delayed central fatigue, may lead to the better performance in the later stage of the high-intensity interval swim.

Our results showed that plasma ammonia and urea were similar between the two trials. In addition, ammonia concentrations remained unchanged after exercise in both trials, indicating that BCAA oxidation was not significantly increased in the AA trial. It is possible that the breakfast and the appropriate warm up before 8 × 50 m swim had already increased plasma ammonia levels. This is in agreement with Peyrebrune et al., who reported that plasma ammonia concentration was decreased after 8 × 50 yards sprint swimming [38]. These results agree with the notion that the major source of ATP turnover comes from aerobic metabolism, rather than adenosine monophosphate (AMP) deamination, towards the end of the repeated swims.

In previous studies we have shown that 0.17 g/kg body weight BCAA, twice of the dosage in the present study, could delay the development of central fatigue and prevent the decline in perceptual-motor functions [22,23]. Although the dosage can be tolerated, the large number of capsules may discourage some athletes from following this supplement regime. The lower dosage of 0.085 g/kg body weight in this study still resulted in an average plasma BCAA concentration of 0.8 mM prior to exercise, which was similar or slightly lower to the previous results [21,22,23]. The combined dosage of citrulline and arginine is 0.1 g/kg body weight, which is in line with previous studies using acute citrulline supplementations [22,23,39].

We specifically use the acute supplementation protocol for the purpose of creating higher plasma BCAA concentration during exercise to compete with free tryptophan in cross blood brain barrier. The longer-term of BCAA supplementation would not lead to significantly higher plasma concentrations compared to the acute regimen. In addition, it has been shown that acute supplements of citrulline-malate or other nitrate sources 40 min to 2.5 h prior to exercise could improve performance and lead to lower steady-state VO_2_ at the same intensity [40,41,42], while several days of beetroot juice supplementation did not elicit greater improvements [40]. Therefore, the acute supplementation regime used in this study would be sufficient for the ergogenic effect.

HIIT has been reported to elicit similar, or even better effects on performance compared to high-volume lower-intensity training in competitive swimmers [6,43]. The shorter duration and lower training volume make HIIT ideal for most athletes and general populations who are living on a tight schedule. Most HIIT in swimming includes repeated sprints of 10 to 30 s duration with resting intervals of 2 to 5 min [44]. Our study adopted this protocol so that the results can be applied to practical training situations. It appears that our participants can maintain speed throughout the present protocol with 3-min recovery between each lap, whereas those with shorter (60 s) recovery periods resulted in significant decrements in performance toward the end [45]. Plasma lactate values in the present study are in line with others using similar protocols in swimmers [37,46], and similar to that after a 100 m swimming competition [47].

Swimming velocity is the product of stroke rate, the number of stroke cycle per min, and stroke length, the distance travelled with each stroke cycle [48]. Elite swimmers usually increase stroke rate, while ignoring stroke length, in order to increase or maintain speed when fatigue is accumulating [49]. In the present study, the participants in the AA trial swam significantly faster in the first and second lap with a trend of higher stroke rate. It is possible that the supplements provided higher central drive in the early stages of 8 × 50 m swim. In the seventh lap, the stroke rate is similar between the two trials. Therefore, the faster speed may result from the longer stroke length, indicating higher muscle strength or technical efficiency in the AA trial during the lap. In the third and fourth laps, the participants swam the fastest, while using the lowest stroke count. This suggests that the participants swam with the highest efficiency in the middle of the 8 × 50 m test.

One of the limitations to this study is the low energy provided in the standard meals during the two days prior to the trial. The meal boxes provided were similar to the participants’ usual diet, both in terms of energy content and food choice. The participants may have had relatively low muscle glycogen levels on the morning of the trials. However, the breakfast before the trial, containing 1.0 g/kg body weight carbohydrate, would ensure the euglycemic state throughout the test. Another limitation is the relatively short exercise time. Whether the ergogenic effect shown in this study can be extended to the entire training period requires further investigation. The lack of data in oxygen consumption and phosphocreatine concentration in the muscle also prevented us from gaining further understanding of the mechanisms.

## 5. Conclusions

The present results showed that BCAA, arginine, and citrulline allowed young participants to swim faster in a 8 × 50 m high-intensity interval protocol while feeling the same level of effort. Future research may focus on the modifications in training load to utilize the enhanced physiological and psychological mechanisms associated with these supplements.

## Figures and Tables

**Table 1:** Basic characteristics of participants.

Gender *	Age (Year)	Height (m)	Weight (kg)	Body Mass Index	Best Style	Personal Best ^†^ 50 m (s)	Personal Best ^†^ 100 m (s)
M	15	1.78	67.1	21.18	Backstroke	29.8	62.2
M	17	1.78	71.1	22.44	Breaststroke	31.2	69.1
M	17	1.77	70.2	22.41	Front crawl	25.7	54.9
M	17	1.78	70.5	22.25	Front crawl	25.6	52.7
M	15	1.69	53.6	18.77	Front crawl	27.3	56.2
M	14	1.68	52.6	18.64	Butterfly	28.9	57.3
M	16	1.69	68.8	24.09	Butterfly	26.8	57.3
M	14	1.71	61.1	20.90	Front crawl	26.2	54.8
Mean ^‡^	15.6	1.74	64.4	21.33			
SD ^‡^	1.3	0.05	7.6	1.89			
F	16	1.46	46.3	21.72	Front crawl	29.7	65.2
F	15	1.59	45.4	17.96	Front crawl	29.8	66.1
F	17	1.60	51.4	20.08	Front crawl	28.2	59.9
F	16	1.64	60.9	22.64	Front crawl	28.0	61.2
F	15	1.53	44.1	18.84	Front crawl	29.5	61.1
F	16	1.58	56.5	22.63	Butterfly	31.3	69.2
F	16	1.65	60.9	22.37	Butterfly	33.5	71.8
F	14	1.60	66.6	26.02	Butterfly	31.7	68.9
Mean ^§^	15.6	1.58	54.0	21.53			
SD ^§^	0.9	0.06	8.4	2.55			

* M: male; F: female; ^†^ personal best record in their respective best style; ^‡^ mean and standard deviation of male participants; ^§^ mean and standard deviation of female participants.

**Table 2:** Biochemical parameters and ratings of perceived exertion at the baseline, before, and after 8 × 50 m test in the in the AA (amino acids supplemented) and PL (placebo) trials.

	Trial	Baseline	Pre-Ex	Post-Ex
NO_x_ (μM)	AA	11.61 ± 4.94	31.65 ± 29.56	23.06 ± 15.28
	PL	12.68 ± 7.42	25.84 ± 29.39	18.36 ± 11.71
NH_3_ (μM)	AA	115.20 ± 103.49	123.72 ± 66.29	123.86 ± 65.35
	PL	116.59 ± 40.69	109.02 ± 50.89	114.81 ± 50.31
Urea (mM)	AA	4.64 ± 0.77	5.38 ± 0.90	5.11 ± 0.91
	PL	4.77 ± 0.65	5.01 ± 0.87	4.63 ± 0.73
Lactate (mM)	AA	1.36 ± 0.56	1.91 ± 0.59	14.75 ± 4.43 *^,†^
	PL	1.43 ± 0.34	1.71 ± 0.38	14.12 ± 2.79 *^,†^
Glycerol (μM)	AA	46.00 ± 21.00	81.89 ± 28.83 *	240.89 ± 96.61 *^,†^
	PL	44.63 ± 25.27	71.35 ± 26.45	227.44 ± 58.36 *^,†^
NEFA (mM) ^1^	AA	0.49 ± 0.27	0.42 ± 0.16	0.22 ± 0.09 ^†^
	PL	0.44 ± 0.25	0.38 ± 0.10	0.20 ± 0.08 ^†^
RPE ^2^	AA	9.1 ± 2.4	15.0 ± 1.8 *	17.2 ± 2.3 *
	PL	9.7 ± 2.4	14.8 ± 2.2 *	17.3 ± 1.8 *

^1^ NEFA. non-esterified fatty acids; ^2^ RPE: ratings of perceived exertion; * Significantly different from the baseline in the same trial, *p* < 0.001; ^†^ Significantly different from Pre-Ex in the same trial, *p* < 0.05. Pre-Ex: before 8 × 50 m swim test; Post-Ex: immediately after 8 × 50 m swim test.

**Table 3:** Stroke rate and stroke count in the 8 × 50 m high-intensity interval swimming test in the AA and PL trials.

Trial		Lap								
		1	2	3	4	5	6	7	8	Mean
Stroke rate ^1^ (count/min)	PL	70.6 ± 19.5	71.2 ± 19.3	72.3 ± 19.0	71.4 ± 20.2	70.2 ± 18.7	69.2 ± 18.5	69.4 ± 18.2	69.4 ± 18.3	70.5 ± 19.0
	AA	74.5 ± 18.5	76.0 ± 18.3	72.7 ± 19.5	72.4 ± 18.4	70.7 ± 16.5	71.4 ± 18.0	70.1± 17.4	71.9 ± 18.2	72.7 ± 18.1
Stroke count ^2^	PL	30.7 ± 9.0 ^a,b^	30.6 ± 9.5 ^a,b^	30.8 ± 9.1 ^a^	30.8 ± 9.2 ^a,b^	31.1 ± 9.4 ^a,b^	31.7 ± 9.5 ^a,b^	31.6 ± 9.4 ^a,b^	31.9 ± 9.0 ^b^	31.1 ± 9.3
(count/lap)	AA	31.1 ± 8.5 ^a,b^	30.6 ± 8.2 ^a,b^	30.6 ± 8.2 ^a^	31.1 ± 8.6 ^a^	31.2 ± 8.3 ^a,b^	31.3 ± 8.1 ^a,b^	31.5 ± 8.4 ^a,b^	32.1 ± 8.4 ^b^	31.2 ± 8.3

^1^ Trial effect *p* = 0.102, lap effect *p* = 0.489, interaction effect *p* = 0.513; ^2^ Trial effect *p* = 0.910, lap effect *p* < 0.001, interaction effect *p* = 0.643; The values with different superscripts (a, b) were significantly different within the same trial.
